# Human Mesenchymal Stem Cells Derived from the Placenta and Chorion Suppress the Proliferation while Enhancing the Migration of Human Breast Cancer Cells

**DOI:** 10.1155/2022/4020845

**Published:** 2022-11-11

**Authors:** Sarawut Sirithammajak, Sirikul Manochantr, Chairat Tantrawatpan, Duangrat Tantikanlayaporn, Pakpoom Kheolamai

**Affiliations:** ^1^Center of Excellence in Stem Cell Research and Innovation, Faculty of Medicine, Thammasat University, Pathumthani 12120, Thailand; ^2^Division of Cell Biology, Faculty of Medicine, Thammasat University, Pathumthani 12120, Thailand

## Abstract

**Background:**

Breast cancer is the most frequently diagnosed malignancy among women, resulting from abnormal proliferation of mammary epithelial cells. The highly vascularized nature of breast tissue leads to a high incidence of breast cancer metastases, resulting in a poor survival rate. Previous studies suggest that human mesenchymal stem cells (hMSCs) play essential roles in the growth, metastasis, and drug responses of many cancers, including breast cancer. However, hMSCs from different sources may release different combinations of cytokines that affect breast cancer differently.

**Methods:**

In this study, we have isolated hMSCs from the placenta (PL-hMSCs) and the chorion (CH-hMSCs) and determined how these hMSCs affect the proliferation, migration, invasion, and gene expression of two human breast cancer cells, MCF-7 and MDA-MB-231, as well as the possible mechanisms underlying those effects.

**Results:**

The results showed that the soluble factors derived from PL-hMSCs and CH-hMSCs inhibited the proliferation of MCF-7 and MDA-MB-231 cells but increased the migration of MDA-MB-231 cells. The study of gene expression showed that PL-hMSCs and CH-hMSCs downregulated the expression levels of the protooncogene *CyclinD1* while upregulating the expression levels of tumor suppressor genes, *P16* and *P21* in MCF-7 and MDA-MB-231 cells. Furthermore, hMSCs from both sources also increased the expression levels of *MYC, SNAI1*, and *TWIST,* which promote the epithelial-mesenchymal transition and migration of breast cancer cells in both cell lines. The functional study suggests that the suppressive effect of CH-hMSCs and PL-hMSCs on MCF-7 and MDA-MB231 cell proliferation was mediated, at least in part, through IFN-*γ*.

**Conclusions:**

Our study suggests that CH-hMSCs and PL-hMSCs inhibited breast cancer cell proliferation by negatively regulating *CYCLIND1* expression and upregulating the expression of the *P16* and *P21* genes. In contrast, hMSCs from both sources enhanced breast cancer cell migration, possibly by increasing the expression of *MYC, SNAI1,* and *TWIST* genes in those cells.

## 1. Background

The uncontrolled proliferation of mammary epithelial cells causes breast cancer, which is the most prevalent malignant tumor in women [1]. Breast cancer metastasis is common due to the high density of blood and lymphatic vessels in breast tissue, resulting in a low survival rate in patients. Although surgical procedures and systemic treatments, such as hormone therapy, chemoradiation, and immunotherapy, have improved significantly, the overall survival rate of patients with advanced-stage breast cancer remains poor due to resistance to treatment and relapse [2, 3].

Several studies have found that human mesenchymal stem cells (hMSCs) play a crucial role in cancer growth, metastasis, and the response to chemotherapeutic drugs. hMSCs are multipotent stem/progenitor cells found in several tissues, including bone marrow, adipose tissue, umbilical cord, placenta, and chorion [4–7]. hMSCs have been recognized as prospective cell sources for several clinical applications due to their ability to release many beneficial bioactive compounds that reduce inflammation, increase cell viability, and enhance neovascularization [8–10]. Additionally, some sources of hMSC, such as bone marrow and adipose tissues, can be harvested from patients and used for autologous transplantation, thus circumventing the need for immunosuppression [10, 11].

In animal models, hMSCs have also been shown to migrate from the bloodstream to cancerous areas [12–14], where they form cancer-associated fibroblasts (CAFs) that promote cancer growth. CAFs derived from hMSCs released multiple soluble factors that induce tumor neovascularization, increase cancer survival, and enhance metastasis [15–23]. Furthermore, hMSCs are believed to promote tumor growth by suppressing immune surveillance against tumor cells [24]. Despite these data, some studies have shown that hMSCs can also limit the growth and spread of several cancers [25–28]. These discrepancies could be caused by differences in the types of hMSCs and cancer used in these investigations.

Although bone marrow-derived hMSCs (BM-hMSCs) have been widely used in most research and therapeutic applications, their harvesting requires an invasive process and their numbers decrease with age [29, 30]. As a result, hMSCs derived from gestational tissues, such as the placenta and chorion, have been deemed more appropriate for therapeutic applications since they are easily available in high quantities using a noninvasive procedure. However, the bioactive molecules produced by hMSCs originating from gestational tissue and their impact on breast cancer cells are still poorly characterized. Therefore, this study is aimed at investigating the effects of placenta-derived hMSCs (PL-hMSCs) and chorion-derived hMSCs (CH-hMSCs) on proliferation, migration, invasion, and gene expression profiles of two different human breast cancer cells, estrogen receptor-expressing MCF-7 cells and estrogen receptor-negative MDA-MB231 cells.

## 2. Methods

### 2.1. Subjects

This study was approved by the Ethics Committee for Human Research of the Faculty of Medicine of Thammasat University (project number: MTU-EC-DS-2-001/62). The study was carried out according to the ICH-GCP. After labor, healthy women donated their placentas and chorions. All donors signed an informed consent form.

### 2.2. Isolation and Culture of hMSCs

As described in our earlier study [31], the placenta and the chorionic membrane were manually separated, minced into small pieces, and incubated for 30 minutes at 37°C in 0.25% (*w*/*v*) trypsin-EDTA (Invitrogen Corporation, USA). The digested tissues were then washed twice with PBS, resuspended in DMEM +10% (*v*/*v*) Fetal Bovine Serum (FBS) (Lonza, USA), and cultured in 25 cm^2^ flasks (Corning, USA) at 37°C. The medium was changed every three days, and the cells were passaged when their density reached 80% confluence. An inverted microscope (Nikon ECLIPSE Ts2R, Japan) was used to examine and photograph the morphological characteristics of hMSCs.

### 2.3. Culture of Human Breast Cancer Cells

Human breast cancer cells, MCF-7 and MDA-MB231, were purchased from ATCC. MCF-7 is an estrogen receptor-expressing human breast cancer cell line derived from the pleural effusion of a female patient with metastatic (stage IV) breast cancer. MDA-MB231 is a triple negative (ER^−^/PR^−^/EGFR^−^) human breast cancer cell line derived from the pleural effusion of a female patient with metastatic (stage IV) breast cancer. Both cells were cultured in DMEM (Invitrogen Corporation, USA) supplemented with 10% (*v*/*v*) FBS (Lonza, USA) at 37°C. The medium was changed every three days, and the cells were passaged when their density reached 80% confluence.

### 2.4. Immunophenotyping of hMSCs by Flow Cytometry

As described in our previous study [31], the hMSCs were collected and immediately processed for flow cytometric analysis. Approximately 4 × 10^5^ hMSCs (passage 3^rd^–5^th^) were resuspended in 50 *μ*l PBS and incubated for 30 minutes at 4°C in the dark with 10 *μ*l fluorochrome-labeled mouse anti-human monoclonal antibodies: anti-CD45-FITC (BD Pharmingen, USA), anti-CD34-PE (BioLegend, USA), anti-CD90-FITC (AbD Serotec, USA), and anti-CD73-PE (BioLegend, USA). After incubation, the cells were washed twice with PBS and fixed with 1% (*w*/*v*) paraformaldehyde in PBS (Sigma-Aldrich, USA). The flow cytometry was performed on a FACSCalibur™ flow cytometer (Becton Dickinson, USA) using CellQuest™ software.

### 2.5. Osteogenic and Adipogenic Differentiation of hMSCs

As described in our previous study [31], 5 × 10^4^ hMSCs were cultured in NH OsteoDiff® Medium (Miltenyi Biotec, Germany) for osteogenic differentiation. The cells were cultured for 28 days with media replacement every three days. At the end of the culture, the cells were fixed with 4% paraformaldehyde, stained with a 40 mM alizarin red S solution (Sigma-Aldrich, USA) for 20 minutes at room temperature, and viewed under an inverted microscope (Nikon ECLIPSE Ts2R, Japan). For adipogenic differentiation, 5 × 10^4^ hMSCs were cultured in NH AdipoDiff® medium (Miltenyi Biotec, Germany) for 28 days with media replacement every three days. At the end of the culture, the cells were fixed with vaporized 37% formalin for 10 minutes at room temperature, stained with 0.5% (*w*/*v*) oil red O (Sigma-Aldrich, USA) in 6% (*v*/*v*) isopropanol for 20 minutes at room temperature and observed by light microscopy.

### 2.6. Preparation of hMSC-Conditioned Medium

As described in our previous study [31], 7 × 10^5^ hMSCs were initially expanded in DMEM +10% FBS until their density reached 90% confluence. The medium was then removed, the cells were rinsed twice with 10 ml sterile PBS, fresh 15 ml DMEM +10% FBS medium was added, and the cells were incubated for another 24 hours. The conditioned medium (CM) was collected after incubation and centrifuged at 1,000 g for 10 minutes at 4°C before being filtered through a 0.45 *μ*m syringe filter (Corning, USA). Finally, the filtered CM was lyophilized and diluted to the appropriate concentrations.

### 2.7. Effects of hMSC-Derived Soluble Factors on the Proliferation of Breast Cancer Cells

5 × 10^3^ MCF-7 or MDA-MB231 cells were seeded into each well in the 96-well plate (SPL Life Sciences, Korea) in DMEM +10% FBS and cultured for 12 hours. At this stage, the media were replaced with 25%, 50%, and 75% (*v*/*v*) hMSC-CM in DMEM +10% FBS. To avoid the nutrient depletion effect in the groups treated with hMSC-CM, various percentages of hMSC-CM were prepared by diluting 10-fold concentrated hMSC-CM in DMEM +10% FBS. MCF-7 or MDA-MB231 cultured in DMEM +10% FBS served as controls. Cell number was measured by an MTT assay (Sigma-Aldrich, USA) every 24 hours for 5 consecutive days. At the time of measurement, the media were removed, 100 *μ*l 2 mg/ml MTT solution was added, and the cells were incubated at 37°C for 3 hours. After incubation, the solution in each well was removed, 100 *μ*l dimethyl sulfoxide (DMSO; VWR BDH Chemicals, France) was added and the absorbance was measured at 570 nm using a microplate reader (BioTex, USA).

### 2.8. In Vitro Migration Assay

The migration assay was performed using a 24-well transwell chamber (Corning, USA) with an 8 *μ*m pore polyester membrane insert. 5 × 10^4^ MDA-MB-231 cells were seeded in transwell inserts containing DMEM +2% FBS and incubated at 37°C for 12 hours to allow cell adhesion. The following days, the inserts containing MDA-MB-231 cells were placed in an individual well of the 24-well plate containing 600 *μ*l of 25%, 50%, and 75% (*v*/*v*) hMSC-CM in DMEM +10% FBS. After culture for 24 hours, the cells that migrated to the other side of the transwell insert were stained with 0.5% (*w*/*v*) Hoechst 33342 in PBS (Sigma-Aldrich, USA) and counted from ten randomly selected microscopic fields. MDA-MB-231 cells cultured with DMEM +30% FBS in the lower chamber served as controls.

### 2.9. In Vitro Invasion Assay

Similar to the migration assay, 5 × 10^4^ MDA-MB-231 cells were resuspended in DMEM +2% FBS, seeded in transwell inserts coated with 0.3 mg/ml Matrigel™ (Corning, USA), and incubated at 37°C for 12 hours to allow cell adhesion. In the following days, the inserts containing MDA-MB-231 cells were placed in an individual well of the 24-well plate (SPL Life Sciences, Korea) containing 5 × 10^4^ hMSCs in 600 *μ*l DMEM +10% FBS, and the cells were cultured for another 24 hours. At this stage, the transwell inserts were washed, fixed with 2% (*v*/*v*) paraformaldehyde in PBS, stained with 0.5% (*w*/*v*) Hoechst 33342 in PBS (Sigma-Aldrich, USA), and observed under a fluorescence microscope (Nikon ECLIPSE Ts2R-FL, Nikon, Japan) to determine the number of invasive cells. MDA-MB-231 cells cultured with DMEM +30% FBS in the lower chamber served as controls.

### 2.10. Gene Expression Study by Quantitative Real-Time PCR (qRT-PCR)

As described in our previous study [31], TRIzol® reagent (Invitrogen Corporation, USA) was used to separate total RNA from cells. The SuperScript™ III Reverse Transcriptase (Invitrogen Corporation, USA) was used to generate cDNA from 2 *μ*g RNA according to the manufacturer's instruction. For qRT-PCR, an Applied Biosystems MicroAmp® quick optical 96-well reaction plate was used. cDNA was mixed with 0.45 *μ*m forward and reverse primers and SYBR® Green PCR Master Mix (Applied Biosystem, USA). The plate was then sealed with a MicroAmp® transparent adhesive film (Applied Biosystem, USA). The following procedure was used for PCR: initial denaturation at 95°C for 10 minutes, followed by 40 cycles of denaturation (95°C, 10 seconds), annealing (60°C, 10 seconds), and extension (72°C, 40 seconds) using an Applied Biosystems 7500 Fast Real-Time PCR system. 7500 software version 2.0.5 (Applied Biosystem, USA) was used to calculate the relative abundance of a target gene by normalizing with glyceraldehyde-3-phosphate dehydrogenase (*GAPDH*). The primer sequences are listed in Table 1.

### 2.11. Statistical Analysis

The mean and standard error of the mean (SEM) was used to present the data. The significance of the differences between the observed data was determined using unpaired student's *t*-test. Statistical significance was defined as a *P* value of less than 0.05.

## 3. Results

### 3.1. Characteristics of hMSCs Derived from the Placenta and Chorion

**Figure 1:**
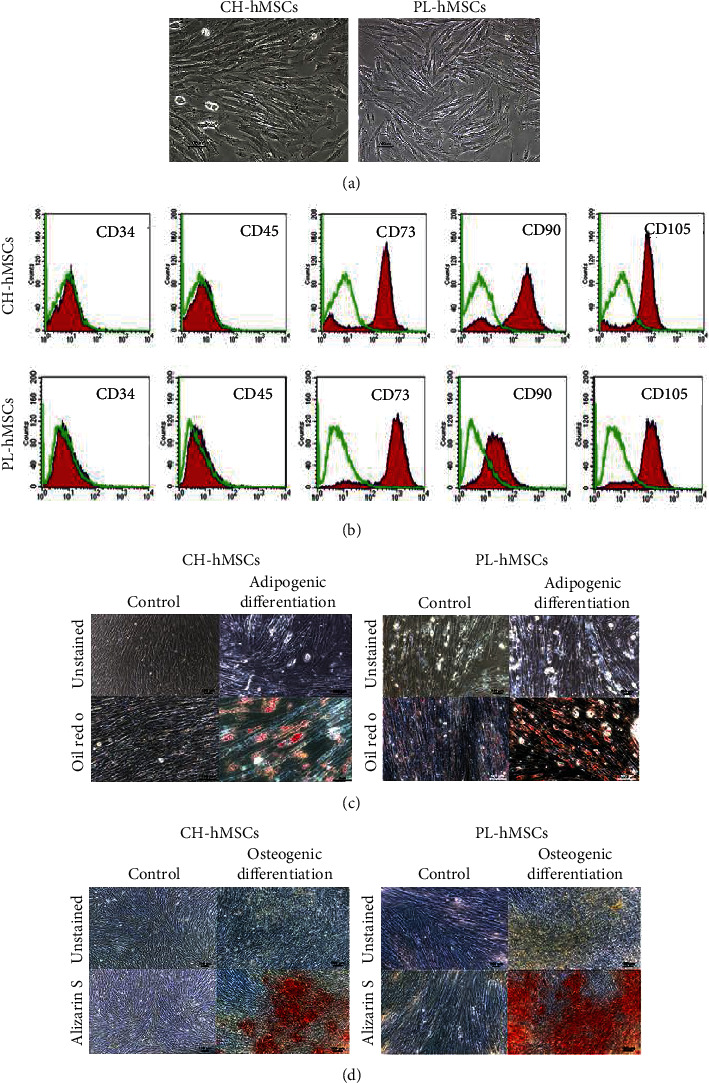
Characteristics of CH-hMSCs and PL-hMSCs. (a) Fibroblast-like morphology of CH-hMSCs and PL-hMSCs (scale bar: 100 *μ*m). (b) Immunophenotypes of CH-hMSCs and PL-hMSCs as determined by flow cytometry. (c) Adipogenic differentiation of CH-hMSCs and PL-hMSCs as determined by oil red O staining (scale bar: 100 *μ*m). (d) Osteogenic differentiation of CH-hMSCs and PL-hMSCs as determined by alizarin red S staining (scale bar: 100 *μ*m).

The placenta-derived hMSCs (PL-hMSCs) and chorion-derived hMSCs (CH-hMSCs) exhibited typical characteristics of hMSCs according to the minimal criteria suggested by the international society for cell therapy [32]. The hMSCs of both sources showed a fibroblast-like morphology (Figure 1(a)), expressed typical surface markers of hMSCs (positive for CD73, CD90, and CD105 and negative for hematopoietic markers, CD34, and CD45; Figure 1(b)), and could differentiate to adipocytes and osteocytes as determined by oil red O staining and alizarin red S staining, respectively (Figures 1(c) and 1(d)).

### 3.2. The Expression Levels of Cytokine Genes in hMSCs Derived from the Placenta and Chorion

**Figure 2:**
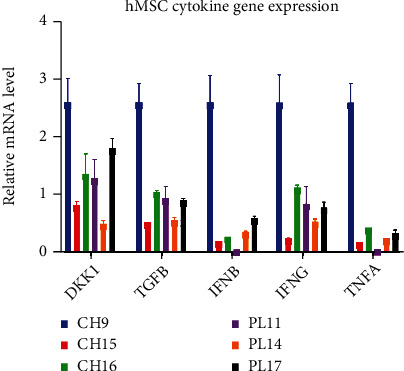
The expression levels of cytokine genes in CH-hMSCs and PL-hMSCs. The expression levels of the cytokine genes were determined by qRT-PCR and standardized with the expression level of *GAPDH* of the same sample. CH-hMSCs were derived from 3 donors (CH9, CH15, and CH16) and PL-hMSCs were derived from 3 donors (PL11, PL14, and PL17). Data are presented as mean ± SEM of three experiments. Abbreviations: DKK1: dickkopf WNT signaling pathway inhibitor 1, TGF-*β*: transforming growth factor-*β*, IFN-*β*: interferon-*β*, IFN-*γ*: interferon-*γ*, TNF-*α*: tumor necrosis factor-*α*.

The expression levels of 5 cytokine genes commonly involved in cancer growth [33–35], including Dickkopf WNT signaling pathway inhibitor 1 (*DKK1*), transforming growth factor-*β* (*TGF-β*), interferon-*β* (*IFN-β*), interferon-*γ* (*IFN-γ*), and tumor necrosis factor-*α* (*TNF-α*) in CH-hMSCs and PL-hMSCs were determined by quantitative real-time PCR. The results showed that the CH-hMSCs and PL-hMSCs derived from most donors expressed high levels of *DKK1, TGF-β*, and *IFN-γ* (Figure 2). Although CH-hMSCs and PL-hMSCs also expressed *IFN-β* and *TNF-α* genes, their expression levels were lower than those of *DKK1, TGF-β*, and *IFN-γ* (Figure 2).

### 3.3. The Effect of hMSC-Derived Soluble Factors on the Proliferation of Human Breast Cancer Cells

**Figure 3:**
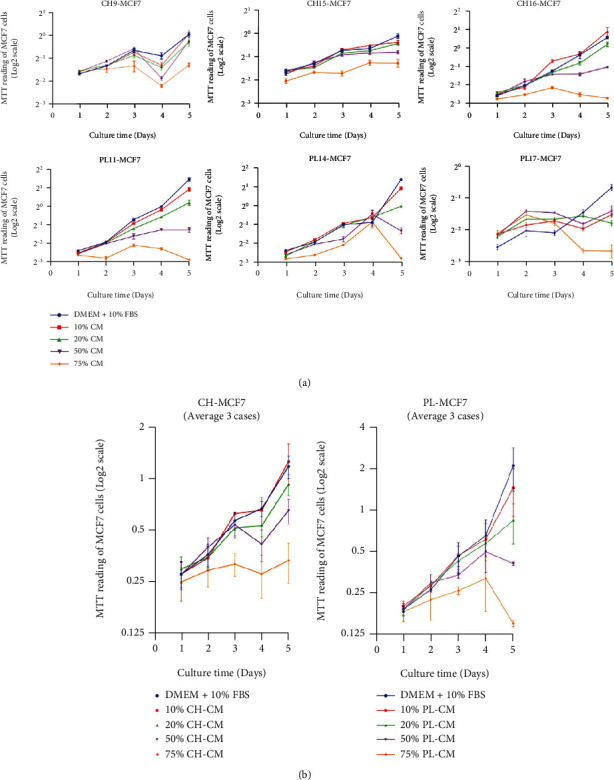
Effect of soluble factors derived from CH-hMSCs and PL-hMSCs on the proliferation of human breast cancer MCF-7 cells. (a) Graphs show the growth kinetics of MCF-7 cells cultured in 10%, 25%, 50%, and 75% conditioned media derived from CH-hMSCs (CH-CM) and PL-hMSCs (PL-CM) determined by the MTT assay. Data are presented as 6 of four experiments. MCF-7 cultured in DMEM medium +10% FBS served as controls. CH-CMs were prepared from 3 CH-hMSCs (CH9, CH15, and CH16) and PL-CMs were prepared from 3 PL-hMSCs (PL11, PL14, and PL17). (b) Graphs show the growth kinetic of MCF-7 cells cultured in 10%, 25%, 50%, and 75% CH-CM (average from CH9, CH15, to CH16) and PL-CM (average from PL11, PL14, to PL17). Data are presented as mean ± SEM of three hMSC-CMs. MCF-7 cultured in DMEM medium +10% FBS served as controls. ^∗^*P* < 0.05 vs. control.

To investigate the effect of hMSC-derived soluble factors on the proliferation of breast cancer cells, 10% (*v*/*v*) -75% (*v*/*v*) conditioned media derived from PL-hMSCs and CH-hMSCs were used to culture two human breast cancer cell lines, the estrogen receptor-expressing MCF-7 cells and the estrogen receptor-negative MDA-MB231 cells, for five days. The results showed that the condition media derived from PL-hMSCs (PL-CM) and CH-hMSCs (CH-CM) significantly reduced MCF-7 proliferation in a dose-dependent manner (Figures 3(a) and 3(b)). The inhibitory effect of PL-CM and CH-CM on MCF-7 and MDA-MB231 cell proliferation was initially observed at 24 hours and was maintained until the end of the culture (Figures 3(a) and 3(b)). The PL-CM and CH-CM derived from 3 different donors (PL11, PL14, and PL17 for PL-CM and CH9, CH1,5 and CH16 for CH-CM) have the same levels of inhibitory effect on the MCF-7 cell proliferation (Figure 3(a)).

**Figure 4:**
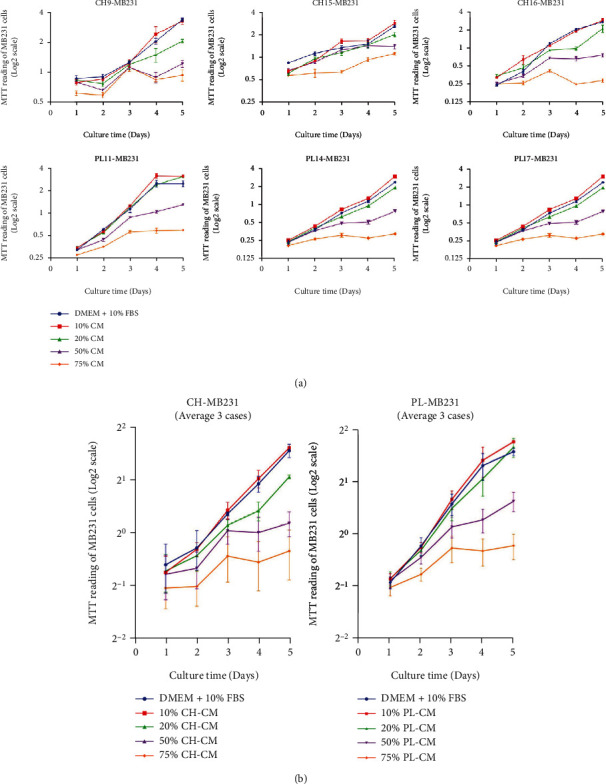
Effect of soluble factor derived from CH-hMSCs and PL-hMSCs on the proliferation of human breast cancer MDA-MB231 cells. (a) Graphs show the growth kinetics of MDA-MB231 cells cultured in 10%, 25%, 50%, and 75% conditioned medium derived from CH-hMSCs (CH-CM) and PL-hMSCs (PL-CM) determined by the MTT assay. Data are presented as mean ± SEM of four experiments. MDA-MB231 cultured in DMEM medium +10% FBS served as controls. CH-CMs were prepared from 3 CH-hMSCs (CH9, CH15, and CH16), and PL-CMs were prepared from 3 PL-hMSCs (PL11, PL14, and PL17). (b) Graphs show the growth kinetics of MDA-MB231 cells cultured in 10%, 25%, 50%, and 75% CH-CM (average from CH9, CH15, to CH16) and PL-CM (average from PL11, PL14, to PL17). Data are presented as mean ± SEM of three hMSC-CMs. MDA-MB231 cultured in DMEM medium +10% FBS served as controls. ^∗^*P* < 0.05 vs. control.

Similar to the effect on MCF-7, PL-CM and CH-CM significantly reduced MDA-MB231 proliferation in a dose-dependent manner (Figures 4(a) and 4(b)). The inhibitory effect on MDA-MB231 cell proliferation was observed at 24 hours and was maintained throughout the culture period (Figures 4(a) and 4(b)). The PL-CM and CH-CM derived from 3 distinct donors also have the same levels of inhibitory effect on the MDA-MB231 cell proliferation (Figure 4(a)).

### 3.4. The Effect of hMSC-Derived Soluble Factors on the Migration of MDA-MB231 Cells

**Figure 5:**
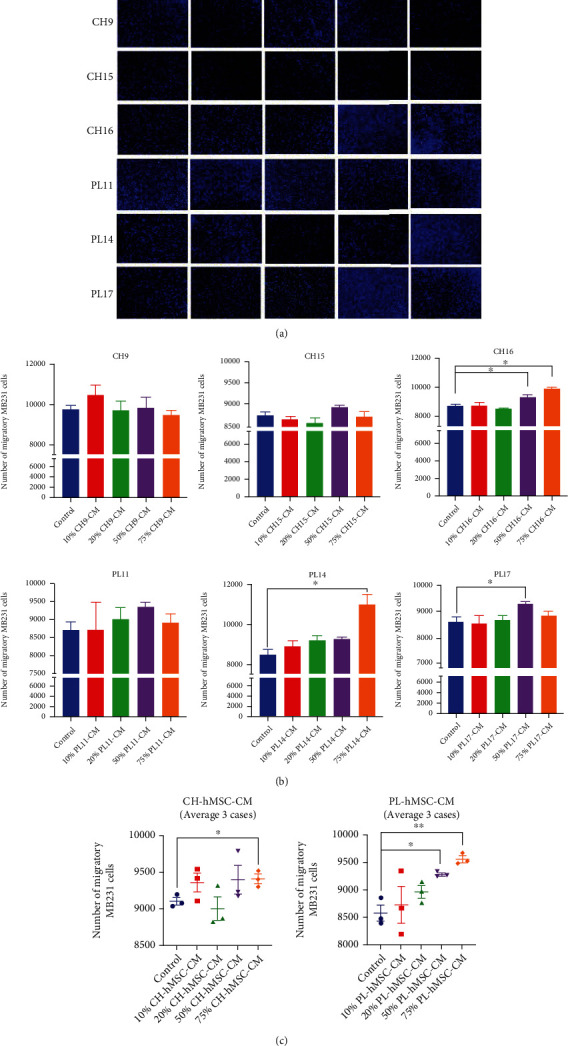
The effect of soluble factors on the migration of human breast cancer MDA-MB231 cells. (a) Representative micrographs of migratory MDA-MB231 cells induced by 10%, 25%, 50%, and 75% conditioned medium derived from CH-hMSCs (CH-CM) PL-hMSCs (PL-CM) determined by the transwell migration assay. CH-CMs were prepared from 3 CH-hMSCs (CH9, CH15, and CH16), and PL-CMs were prepared from 3 PL-hMSCs (PL11, PL14, and PL17). MDA-MB231 cells induced by DMEM medium +30% FBS served as controls. (b) Graphs show the number of migratory MDA-MB231 cells induced by 10%, 25%, 50%, and 75% conditioned media derived from CH-CM and PL-CM determined by transwell migration assay. Data are presented as mean ± SEM of three experiments. CH-CMs were prepared from 3 CH-hMSCs (CH9, CH15, and CH16), and PL-CMs were prepared from 3 PL-hMSCs (PL11, PL14, and PL17). MDA-MB231 cells induced by DMEM medium +30% FBS served as controls. ^∗^*P* < 0.05 vs. control, ^∗∗^*P* < 0.01 vs. control. (c) Graphs show the number of migratory MDA-MB231 cells induced by 10%, 25%, 50%, and 75% conditioned media derived from CH-hMSC-CM (average of CH9, CH15, and CH16) and PL-hMSC-CM (average of PL11, PL14, and PL17). Data are presented as mean ± SEM of three hMSC-CMs. MDA-MB231 induced by DMEM medium +30% FBS served as controls. ^∗^*P* < 0.05 vs. control, ^∗∗^*P* < 0.01 vs. control.

To study the effect of hMSC-derived soluble factors on the migration of breast cancer cells, MDA-MB231 cells were induced to migrate through transwell with 8 *μ*m pore using 10% (*v*/*v*) -75% (*v*/*v*) conditioned media derived from PL-hMSCs and CH-hMSCs. The numbers of MDA-MB231 cells that migrate through the other side of the membrane were determined after 24 hours of induction. 75% CH-CM (*v*/*v*) significantly enhanced MDA-MB231 cell migration compared with controls induced by DMEM +30% FBS (9,407 ± 65.6 cells vs. 9,097 ± 49 cells, *P* < 0.05) (Figures 5(a)–5(c)). Similar to the CH-CM, 50% and 75% PL-CM also significantly enhanced MDA-MB231 cell migration compared with controls (8,969 ± 114 cells vs. 8,578 ± 141.5 cells, *P* < 0.01 and 9,573 ± 160.4 cells vs. 8,578 ± 141.5 cells, *P* < 0.01, respectively) (Figures 5(a)–5(c)). These results suggest that the soluble factors derived from CH-hMSCs and PL-hMSCs enhanced the migratory capacity of MDA-MB231 cells in a dose-dependent manner. It is worth noting that there are variations in migration-enhancing ability among PL-hMSCs and CH-hMSCs derived from different donors (Figures 5(a) and 5(b)). Due to the very low migratory and invasive capacity of MCF-7 cells in our study, which is also described elsewhere [36–38], we only use MDA-MB231 cells for our migration and invasion assays.

### 3.5. The Effect of hMSC-Derived Soluble Factors on the Invasion of MDA-MB231 Cells

**Figure 6:**
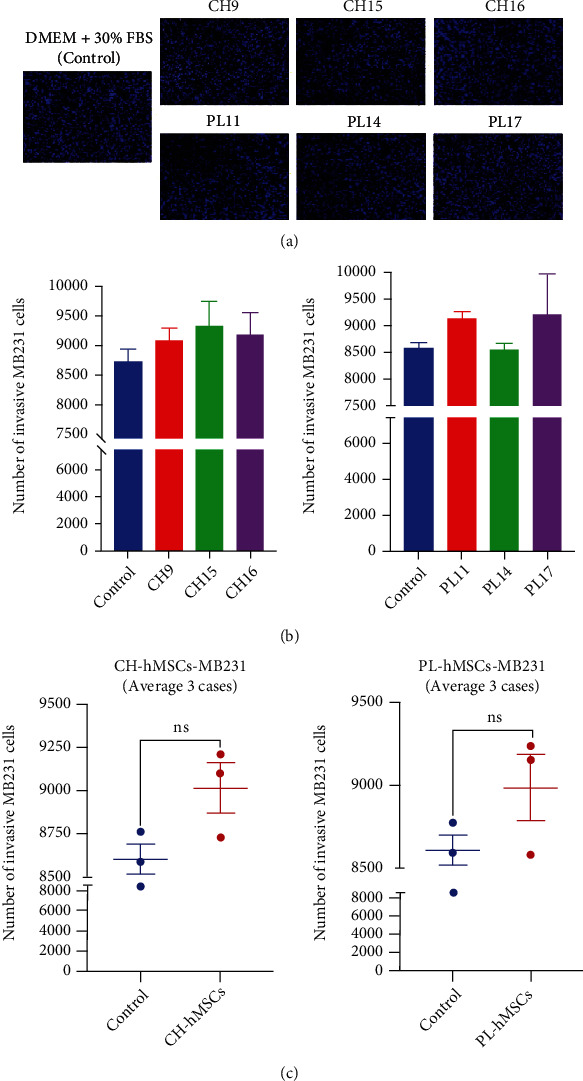
The effect of soluble factors derived from CH-hMSCs and PL-hMSCs on the invasion of human breast cancer MDA-MB231 cells. (a) Representative micrographs of the invasive MDA-MB231 cells after coculture with CH-hMSCs and PL-hMSCs as determined by transwell invasion assay. CH-hMSCs were derived from three donors (CH9, CH15, and CH16), and PL-hMSCs were derived from three donors (PL11, PL14, and PL17). MDA-MB231 cells induced by DMEM medium +30% FBS served as controls. (b) Graphs show the number of invasive MDA-MB231 cells after being cocultured with CH-hMSCs and PL-hMSCs as determined by transwell invasion assay. Data are presented as mean ± SEM of three experiments. CH-hMSCs were derived from three donors (CH9, CH15, and CH16), and PL-hMSCs were derived from three donors (PL11, PL14, and PL17). MDA-MB231 cells induced by DMEM medium +30% FBS served as controls. (c) Graphs show the number of invasive MDA-MB231 cells after being induced by cocultured with CH-hMSCs (average from CH9, CH15, and CH16) and PL-hMSCs (average from PL11, PL14, and PL17). Data are presented as mean ± SEM of three hMSCs. MDA-MB231 cells cultured in DMEM medium +30% FBS served as a control.

To study the effect of hMSC-derived soluble factors on the invasion of breast cancer cells, MDA-MB231 cells were cocultured with CH-hMSCs and PL-hMSCs using the Matrigel-coated transwells with 8 *μ*m pore. The number of MDA-MB231 cells that invade Matrigel and move through the other side of the membrane was determined after 24 hours of coculture (Figure 6(a)). Unlike the migration assay, CH-hMSCs and PL-hMSCs did not significantly affect the number of invading MDA-MB231 cells compared with controls that were induced by DMEM +30% FBS (9,019 ± 146.2 cells vs. 8,607 ± 86.7 cells and 8,980 ± 202.6 cells vs. 8,607 ± 86.7 cells, respectively) (Figure 6(a)–6(c)). These results suggest that although the soluble factors derived from CH-hMSCs and PL-hMSCs enhanced the migratory ability of MDA-MB231 cells, they did not significantly affect the invasion of MDA-MB231 cells.

### 3.6. The Effect of hMSC-Derived Soluble Factors on the Gene Expression of Human Breast Cancer Cells

To study the effect of hMSC-derived soluble factors on the expression of genes involved in the proliferation and migration of human breast cancer cells, the MCF-7, and MDA-MB231 cells were cocultured with CH-hMSCs or PL-hMSCs using the transwell system for 24 hours. After coculture, the expression levels of the *Cyclin-D1, P16, P21, P27, MYC, SNAI1,* and *TWIST* genes in MCF-7 and MDA-MB231 cells were determined by qRT-PCR.

**Figure 7:**
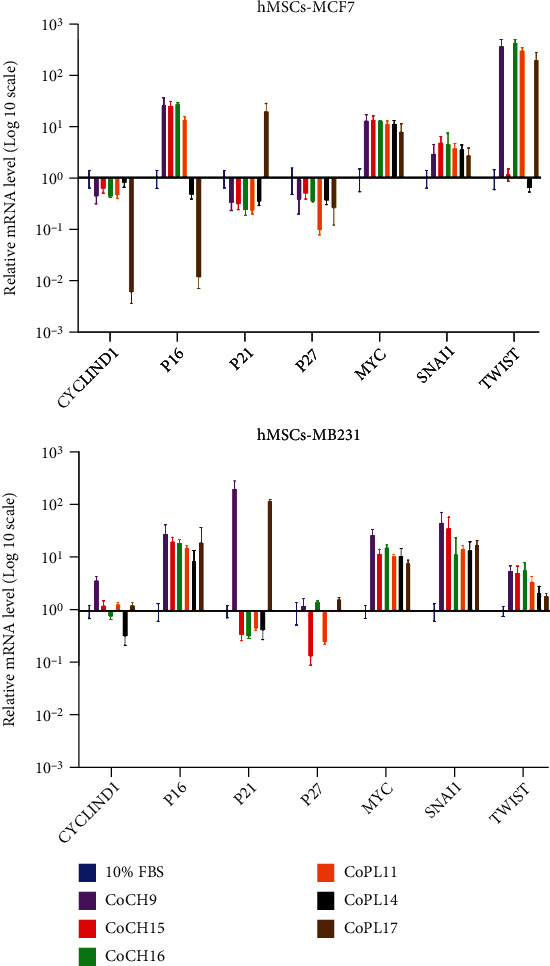
The effect of hMSC-derived soluble factors on the gene expression of human breast cancer MCF-7 and MDA-MB231 cells. Gene expression levels in MCF-7 and MDA-MB231 cells cocultured with CH-hMSCs (coCH) and PL-hMSCs (coPL) using a transwell culture system as determined by qRT-PCR. CH-hMSCs were derived from 3 donors (CH9, CH15, and CH16), and PL-hMSCs were derived from 3 donors (PL11, PL14, and PL17). MCF-7 and MDA-MB231 cells cultured in DMEM medium +10% FBS (10% FBS) served as controls. The relative expression levels of the genes in each sample were standardized with the expression level of the same genes in the control samples. Data are presented as mean ± SEM of three experiments.

The soluble factors derived from most CH-hMSCs and PL-hMSCs downregulated the expression level of the protumorigenic gene *Cyclin-D1* while upregulated the expression level of tumor suppressor gene *P16* in MCF-7 cells (Figure 7 and Supplementary Materials). Likewise, hMSC-derived factors increased the expression level of the *P16* and cyclin-dependent kinase inhibitor *P21* genes in MDA-MB231 cells (Figure 7 and Supplementary Materials). The expression levels of these three cell cycle regulators agreed with the reduction of MCF-7 and MDA-MB231 proliferation after being treated with conditioned media derived from CH-hMSCs and PL-hMSCs. Furthermore, soluble factors derived from CH-hMSCs and PL-hMSCs also increased the expression levels of *MYC, SNAI1,* and T*WIST,* which play critical roles in the epithelial-mesenchymal transition and migration of cancer cells (Figure 7 and Supplementary Materials).

### 3.7. The Effects of *DKK1*, *TGF-β*, and *IFN-γ* Released from hMSCs on the Proliferation of Human Breast Cancer Cells

**Figure 8:**
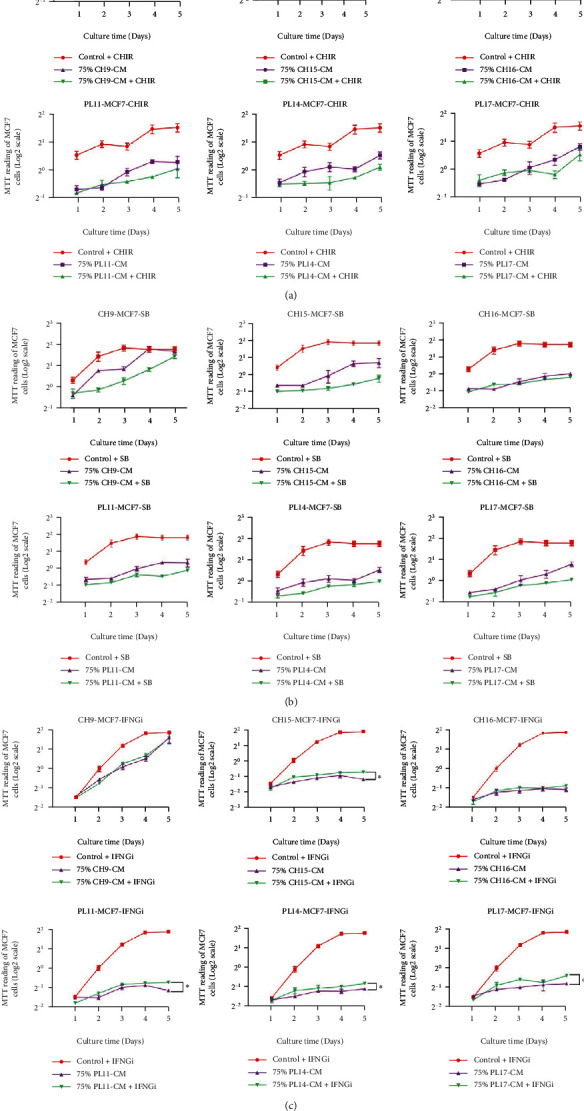
The effect of DKK1, TGF-*β*, and IFN-*γ* on the proliferation of human breast cancer MCF-7 cells in the presence of soluble factors derived from PL-hMSCs and CH-hMSCs. (a) Graphs show the growth kinetics of MCF-7 cells cultured in 75% conditioned medium derived from CH-hMSCs and PL-hMSCs supplemented with the DKK1 antagonist CHIR99021 (75% CH-CM+CHIR and 75% PL-CM+CHIR, respectively) as determined by the MTT assay. Data are presented as mean ± SEM of four experiments. MCF-7 cultured in DMEM medium +10% FBS supplemented with CHIR99021 (control+CHIR) served as controls. (b) Graphs show the growth kinetics of MCF-7 cells cultured in 75% conditioned media derived from CH-hMSC and PL-hMSCs supplemented with TGF-*β* inhibitor SB431542 (75% CH-CM+SB and 75% PL-CM+SB, respectively) as determined by the MTT assay. Data are presented as mean ± SEM of four experiments. MCF-7 cultured in DMEM medium +10% FBS supplemented with SB431542 (control+SB) served as controls. (c) Graphs show the growth kinetics of MCF-7 cells cultured in 75% conditioned media derived from CH-hMSC and PL-hMSCs supplemented with IFN-*γ* neutralizing antibody XMG1.2 (75% CH-CM+IFNGi and 75% PL-CM+IFNGi, respectively) as determined by the MTT assay. Data are presented as mean ± SEM of four experiments. MCF-7 cultured in DMEM medium +10% FBS supplemented with XMG1.2 (control+IFNGi) served as controls. ^∗^*P* < 0.05 between the compared groups. CH-CMs were prepared from 3 CH-hMSCs (CH9, CH15, and CH16), and PL-hMSC-CMs were prepared from 3 PL-hMSCs (PL11, PL14, and PL17).

**Figure 9:**
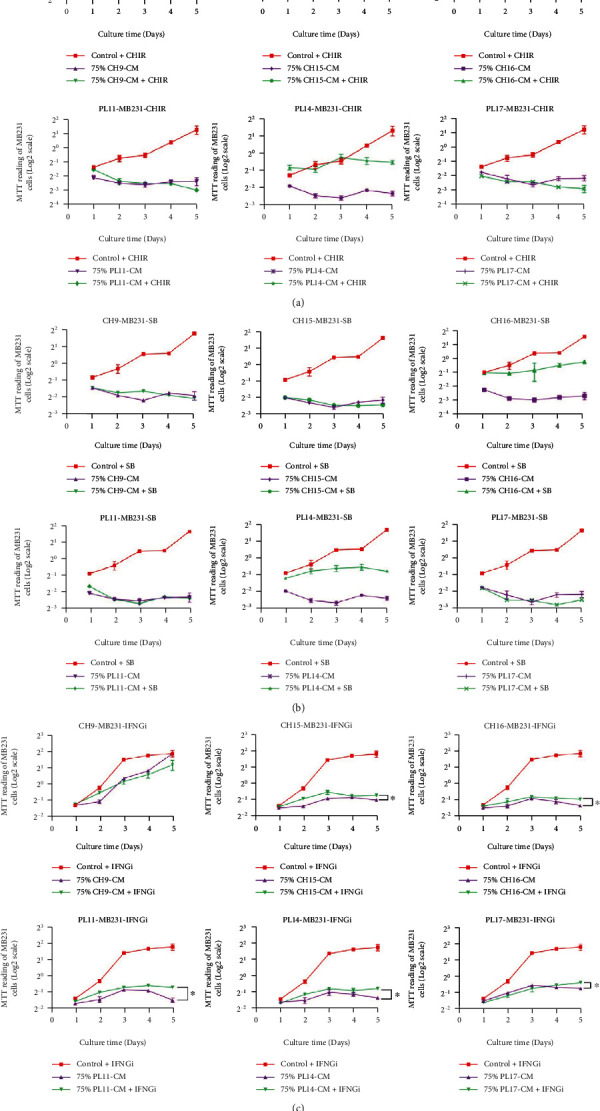
The effect of DKK1, TGF-*β*, and IFN-*γ* on the proliferation of human breast cancer MDA-MB231 cells in the presence of soluble factors derived from PL-hMSCs and CH-hMSCs. (a) Graphs show the growth kinetics of MDA-MB231 cells cultured in 75% conditioned medium derived from CH-hMSCs and PL-hMSCs supplemented with the DKK1 antagonist CHIR99021 (75% CH-CM+CHIR and 75% PL-CM+CHIR, respectively) determined by the MTT assay. Data are presented as mean ± SEM of four experiments. MDA-MB231 cultured in DMEM medium +10% FBS supplemented with CHIR99021 (control+CHIR) served as controls. (b) Graphs show the growth kinetics of MDA-MB231 cells cultured in 75% conditioned media derived from CH-hMSCs and PL-hMSCs supplemented with TGF-*β* inhibitor SB431542 (75% CH-CM+SB and 75% PL-CM+SB, respectively) as determined by the MTT assay. Data are presented as mean ± SEM of four experiments. MDA-MB231 cultured in DMEM medium +10% FBS supplemented with SB431542 (control+SB) served as controls. (c) Graphs show the growth kinetics of MDA-MB231 cells cultured in 75% conditioned media derived from CH-hMSCs and PL-hMSCs supplemented with IFN-*γ* neutralizing antibody XMG1.2 (75% CH-CM+IFNGi and 75% PL-CM+IFNGi, respectively) determined by the MTT assay. Data are presented as mean ± SEM of four experiments. MDA-MB231 cultured in DMEM medium +10% FBS supplemented with XMG1.2 (control+IFNGi) served as controls. ^∗^*P* < 0.05 between the compared groups. CH-CMs were prepared from 3 CH-hMSCs (CH9, CH15, and CH16), and PL-CMs were prepared from 3 PL-hMSCs (PL11, PL14, and PL17).

According to the study of CH-hMSC and PL-hMSC gene expression (Figure 2), we hypothesized that *DKK1, TGF-β*, and *IFN-γ* might be responsible for the inhibitory effects of hMSCs on the proliferation of MCF-7 and MDA-MB231 cells. To investigate this hypothesis, MCF-7 and MDA-MB231 cells were cultured in 75% CH-CM (*v*/*v*) or 75% (*v*/*v*) PL-CM supplemented with 3 *μ*m CHIR99021 (WNT activator and *DKK1* antagonist), 5 *μ*m SB431542 (TGF-*β* inhibitor), and 2 *μ*g XMG1.2 (a neutralizing antibody against IFN-*γ*) for 5 days. The results showed that the addition of CHIR99021 or SB431542 did not decrease the ability of CH-hMSCs and PL-hMSCs to suppress the proliferation of MCF-7 (Figure 8) and MDA-MB231 cells (Figure 9). The results demonstrate that CH-hMSCs and PL-hMSCs did not suppress the proliferation of MCF-7 and MDA-MB231 cells through the action of DKK1 and TGF-*β*. Unlike CHIR99021 and SB431542, XMG1.2 significantly reduced the ability of CH-hMSCs and PL-hMSCs to suppress MCF-7 and MDA-MB231 cell proliferation (Figures 8 and 9). These findings suggest that IFN-*γ* is one of the factors involved in the suppressive effect of CH-hMSCs and PL-hMSCs on MCF-7 and MDA-MB231 cell proliferation.

## 4. Discussion

hMSCs have long been regarded as one of the most promising sources for cell therapy. The capacity of hMSCs to release a wide range of bioactive chemicals that alter various processes, including immune response, cell growth, and neovascularization, is crucial to their therapeutic potential [39]. hMSCs also play a role in cancer progression by releasing angiogenic factors, such as IGF-1, SDF-1, and PDGF, which promote tumor neovascularization, enhance tumor engraftment, and suppress immune responses to cancer cells [16, 17, 19, 20, 22, 24, 40–42]. However, several other studies found that hMSC prevented the growth and spread of several malignant neoplasms in animal models, including colon cancer, hepatocellular carcinoma, and malignant melanoma [25–28]. The variations could be attributed to differences in the sources of hMSCs and types of cancer cells used in the studies. The hMSCs derived from the placenta and chorion (PL-hMSCs and CH-hMSCs) have been considered to be more appropriate sources of hMSCs for therapeutic applications than the commonly used BM-hMSCs, since they may be easily obtained in high quantities using a noninvasive technique. However, the types of bioactive chemicals produced by hMSCs originating from these gestational tissues and their impact on the properties of breast cancer, the most prevalent cancer in women, have yet to be determined.

Our study found that bioactive compounds released from PL-hMSCs and CH-hMSCs inhibited the proliferation of MCF-7 and MDA-MB-231 cells in a dose-dependent manner. Both sources of hMSC suppressed breast cancer cell proliferation in the same way, and there was no difference between hMSCs generated from different donors. According to our study of gene expression, soluble factors derived from CH-hMSCs and PL-hMSCs inhibited the cell proliferation of MCF-7 and MDA-MB-231 by negatively regulating the expression level of the protumorigenic gene *Cyclin-D1* and upregulated the tumor suppressor gene *P16* and the cyclin-dependent kinase inhibitor genes *P21* in these cells. All of these genes have been shown to play a critical role in the onset and progression of breast cancer. The inactivation of p16^INK4a^, a CDK inhibitor that inactivates CDK4/6 and prevents cell cycle progression at the G1/S checkpoint, resulting in the loss of cell cycle control, while dysregulation of the CDK4/6-cyclin D1 complex is a crucial stage in the genesis of breast cancer [43–46].

In contrast to their effect on breast cancer cell proliferation, the soluble factors derived from CH-hMSCs and PL-hMSCs enhanced MDA-MB231 cell migration in a dose-dependent manner. Consistent with this observation, our gene expression study showed that the soluble factors derived from CH-hMSCs and PL-hMSCs upregulated the expression levels of *MYC, SNAI1,* and T*WIST* that promote epithelial to mesenchymal transition and migration of breast cancer cells [47–51]. There are variances in the ability of PL-hMSCs and CH-hMSCs obtained from different donors to enhance MDA-MB231 migration, which could be due to differences in the amount of released cytokines. Interestingly, although both sources of hMSCs increased the migration of MDA-MB231 cells, they did not affect the invasion of MDA-MB231 cells. These findings suggested that the cytokines that cause MDA-MB231 cells to migrate may be different from those that cause them to invade.

According to the study of CH-hMSC and PL-hMSC gene expression, we hypothesized that DKK1, TGF-*β*, and IFN-*γ* might be responsible for the inhibitory effects of hMSCs on the proliferation of MCF-7 and MDA-MB231 cells. Our functional study using CHIR99021, SB431542, and XMG1.2 suggests that CH-hMSCs and PL-hMSCs suppressed MCF-7 and MDA-MB231 cell proliferation, at least in part, through IFN-*γ* rather than DKK1 and TGF-*β*. This result contrasts with a previous study showing that BM-hMSCs promote breast cancer cell proliferation, migration, and invasion under hypoxic conditions by releasing TGF-*β*1 [52]. The discrepancies are most likely due to the different types of hMSCs used in each study. Due to the heterogeneous nature of hMSCs at the time of isolation and subsequent expansion, all sources of hMSCs, including BM-hMSCs, likely consist of several subpopulations that might release different combinations of cytokines and therefore exert different effects on breast cancer cells. Therefore, hMSCs derived from different tissues or those derived from the same tissue from different donors could exhibit distinct characteristics and biological properties, making the outcome of therapy difficult to predict. Consequently, we believe that all hMSCs, regardless of the sources, should be characterized in terms of their cytokine expression before being used for various therapeutic purposes. The knowledge gained from this study could help identify soluble factors derived from hMSCs that could be used to inhibit breast cancer cell growth, so hMSCs derived from many tissue sources or donors could be screened for those therapeutically beneficial factors and, therefore, the suitable MSCs could be selected for treatment.

## 5. Conclusion

Our study suggests that CH-hMSCs and PL-hMSCs inhibited breast cancer cell proliferation by negatively regulating *CyclinD1* expression and upregulating the expression of the *P16* and *P21* genes. The suppressive effect could be mediated, at least in part, by IFN-*γ* released from the hMSCs. On the contrary, hMSCs from both sources enhanced breast cancer cell migration, possibly by increasing the expression of the *MYC, SNAI1*, and *TWIST* genes in these cells. We believe that the findings of this study could be used to design more effective treatments for patients with advanced-stage breast cancer by modulating the interaction between breast cancer cells and hMSCs.

## Figures and Tables

**Table 1:** List of primers.

Genes of interest	Sequences	
	Forward primer (5′→ 3′)	Reverse primer (5′→ 3′)
*SNAI1*	GCGAGCTGCAGGACTCTAAT	GGACAGAGTCCCAGATGAGC
*TWIST1*	GGCTCAGCTACGCCTTCTC	CACGCCCTGTTTCTTTGAAT
*MYC*	TTTCGGGTAGTGGAAAACCA	CAGCAGCTCGAATTTCTTCC
*Cyclin D1 (CCND1)*	CGTGGCCTCTAAGATGAAGG	CTGGCATTTTGGAGAGGAAG
*P16 (CDKN2A)*	GCACCAGAGGCAGTAACCAT	TTACGGTAGTGGGGGAAGG
*P21 (CDKN1A)*	GGAAGACCATGTGGACCTGT	GGATTAGGGCTTCCTCTTGG
*P27 (CDKN1B)*	GGCCTCAGAAGACGTCAAAC	TGCATAATGCTACATCCAACG
*DKK1*	TCCAACGCTATCAAGAACCTG	TGGGACTAGCGCAGTACTCAT
*TNFA*	GGACACCATGAGCACTGAAA	GCCAGAGGGCTGATTAGAGA
*TGFB*	GTGACCTGGCCACCATTC	GTCCTTGCGGAAGTCAATGT
*IFNB*	CATTACCTGAAGGCCAAGGA	CAGCATCTGCTGGTTGAAGA
*IFNG*	GAGTGTGGAGACCATCAAGGA	ATATTGCAGGCAGGACAACC
*GAPDH*	CGAGATCCCTCCAAAATCAA	TTCACACCCATGACGAACAT

## Data Availability

All data generated or analyzed during this study are included in this published article.

## Abbreviations

CH-hMSCs: Chorion-derived human mesenchymal stem cells
PL-hMSCs: Placenta-derived human mesenchymal stem cells
CH-CM: Conditioned medium derived from CH-hMSCs
PL-CM: Conditioned medium derived from PL-hMSCs.

## Additional Points

*Novelty and Impact Statements.* Our study demonstrates that placenta- and chorion-derived hMSCs inhibited the proliferation of human breast cancer cells MCF-7 and MDA-MB-231 by negatively regulating *CYCLIND1* expression and upregulating the expression of the *P16* and *P21* genes. On the contrary, the hMSCs derived from both sources enhanced breast cancer cell migration, possibly by increasing the expression of *MYC*, *SNAI1*, and *TWIST* genes in those cells. The functional study suggests that the suppressive effect of CH-hMSCs and PL-hMSCs on MCF-7 and MDA-MB231 cell proliferation was mediated, at least in part, through IFN-*γ*. The knowledge gained from this research provides a better understanding of the effects of human gestational tissue-derived MSCs on the properties of breast cancer cells involved in their growth and metastasis. This knowledge might be used to improve the strategy of using hMSCs to treat advanced-stage breast cancer.

## Supplementary Materials

Supplementary Materials Table S1. Effect of hMSCs on MCF-7 gene expression.pdf which shows the expression level of genes in MCF-7 cells cocultured with hMSCs. Table S2. Effect of hMSCs on MB231 gene expression.pdf which shows the expression level of genes in MDA-MB231 cells cocultured with hMSCs. Table S3. Effect of hMSCs on MCF7 gene expression raw qRT-PCR data as exported from the Bio-Rad.mgxd file. Table S4. Effect of hMSCs on raw qRT-PCR data for MB231 gene expression exported from the Bio-Rad.mgxd file.
