# Supplementary material for: Human Mesenchymal Stem Cells Derived from the Placenta and Chorion Suppress the Proliferation while Enhancing the Migration of Human Breast Cancer Cells
Source: Stem Cells Int. 2022 Nov 11;2022:4020845. doi: 10.1155/2022/4020845 (PMC9674426; doi:10.1155/2022/4020845)
Supplement: Supplementary Materials — Table S1. Effect of hMSCs on MCF-7 gene expression.pdf which shows the expression level of genes in MCF-7 cells cocultured with hMSCs. Table S2. Effect of hMSCs on MB231 gene expression.pdf which shows the expression level of genes in MDA-MB231 cells cocultured with hMSCs. Table S3. Effect of hMSCs on MCF7 gene expression raw qRT-PCR data as exported from the Bio-Rad.mgxd file. Table S4. Effect of hMSCs on raw qRT-PCR data for MB231 gene expression exported from the Bio-Rad.mgxd file. [file 4020845.f1.zip › Effect of hMSCs on MB231 gene expression raw qRT-PCR data.pdf]

# hMSC-MB231 complete.mgxd

10/22/2022 3:12 PM

## Report Information

**User** : BioRad/admin
**File Name** : hMSC-MB231 complete.mgxd
**File Path** : \\Mac\Home\Desktop\SCI re-submission 2022\Gene expression data
**Report Differs from Last Save** : No

## Gene Study File List

| File Name | File Path | Date Created | Well Group Name | Run Type | Protocol Edited |
|---|---|---|---|---|---|
| 2021-08-14 Experiment5.MB231 target gene part2.pcrd | \\Mac\Home\Desktop | 8/14/2021 5:36:11 PM | All Wells | User-defined | No |
| 2021-08-14 Experiment4.MB231 target gene part1 - cyclin D.pcrd | \\Mac\Home\Desktop | 8/14/2021 5:35:43 PM | All Wells | User-defined | No |
| 2021-11-03 MB231 4R result.pcrd | \\Mac\Home\Desktop | 11/3/2021 8:09:34 PM | All Wells | User-defined | No |
| 2021-10-13 Experiment8 MB231 target gene part3R.pcrd | \\Mac\Home\Desktop | 10/13/2021 7:31:29 PM | All Wells | User-defined | No |

## Study Analysis - Bar Chart

**Analysis Mode** : Normalized expression ( ΔΔCq )
**Chart Data** : Relative to control
**Scaling options** :
**Chart Error** : ± 1.0 SEMs

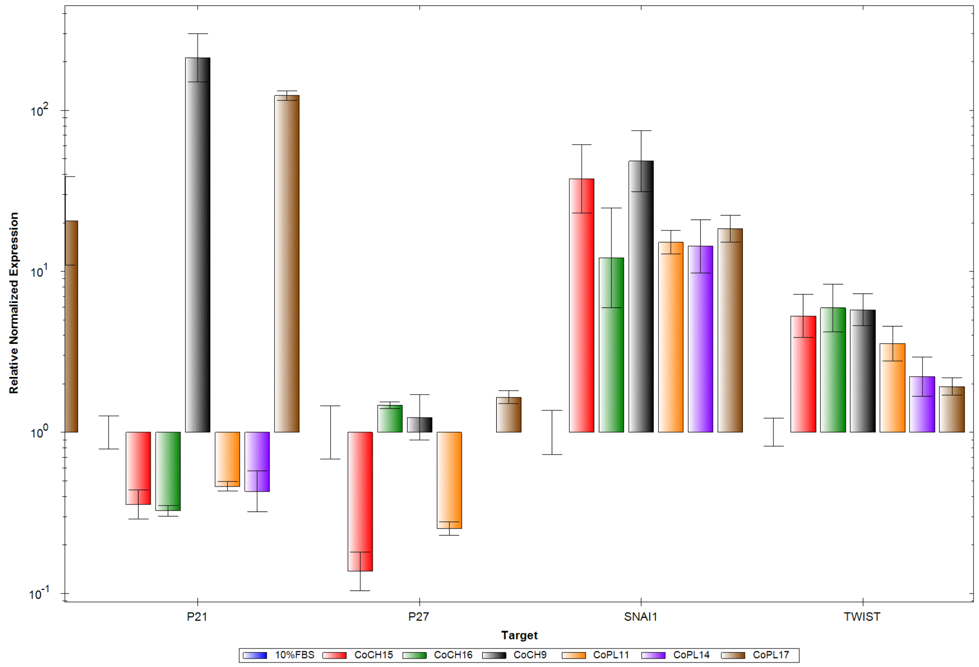


## Target Names

| Name | Full Name | Reference | Auto Efficiency | Efficiency |
|---|---|---|---|---|
| CyclinD1 | CyclinD1 | False | Yes | 100.0% |
| E2F2 | E2F2 | False | Yes | 100.0% |
| MYC | MYC | False | Yes | 100.0% |
| P16 | P16 | False | Yes | 100.0% |

| | | | | |
|---|---|---|---|---|
| P21 | P21 | False | Yes | 100.0% |
| P27 | P27 | False | Yes | 100.0% |
| SNAI1 | SNAI1 | False | Yes | 100.0% |
| TWIST | TWIST | False | Yes | 100.0% |
| GAPDH | GAPDH | True | Yes | 100.0% |

Sample Names

| Name | Full Name | Control |
|---|---|---|
| 10%FBS | 10%FBS | Yes |
| CoCH15 | CoCH15 | No |
| CoCH16 | CoCH16 | No |
| CoCH9 | CoCH9 | No |
| CoPL11 | CoPL11 | No |
| CoPL14 | CoPL14 | No |
| CoPL17 | CoPL17 | No |

Study Analysis - Bar Chart Data

| Target | Sample | Ctrl | Expression | Expression SEM | Corrected Expression SEM | Mean Cq | Cq SEM |
|---|---|---|---|---|---|---|---|
| CyclinD1 | 10%FBS | * | 1.00000 | 0.28976 | 0.28976 | 17.68 | 0.17637 |
| CyclinD1 | CoCH15 | | 1.26531 | 0.28762 | 0.28762 | 17.05 | 0.02575 |
| CyclinD1 | CoCH16 | | 0.78424 | 0.10344 | 0.10344 | 17.86 | 0.18313 |
| CyclinD1 | CoCH9 | | 3.79400 | 0.78806 | 0.78806 | 16.08 | 0.02599 |
| CyclinD1 | CoPL11 | | 1.33768 | 0.09877 | 0.09877 | 16.73 | 0.02018 |
| CyclinD1 | CoPL14 | | 0.32805 | 0.10611 | 0.10611 | 18.25 | 0.05703 |
| CyclinD1 | CoPL17 | | 1.28274 | 0.14276 | 0.14276 | 16.66 | 0.12580 |
| E2F2 | 10%FBS | * | 1.00000 | 0.87750 | 0.87750 | 30.83 | 1.20790 |
| E2F2 | CoCH15 | | 1.71572 | 0.91681 | 0.91681 | 29.77 | 0.69816 |
| E2F2 | CoCH16 | | 0.64584 | 0.02353 | 0.02353 | 31.29 | 0.00964 |
| E2F2 | CoCH9 | | 0.40844 | 0.19194 | 0.19194 | 32.45 | 0.60871 |
| E2F2 | CoPL11 | | 1.91844 | 0.53292 | 0.53292 | 29.36 | 0.38688 |
| E2F2 | CoPL14 | | 1.35467 | 0.45234 | 0.45234 | 29.36 | 0.13257 |
| GAPDH | 10%FBS | * | N/A | N/A | N/A | 32.09 | 0.30945 |
| GAPDH | CoCH15 | | N/A | N/A | N/A | 31.80 | 0.26694 |
| GAPDH | CoCH16 | | N/A | N/A | N/A | 31.91 | 0.05167 |
| GAPDH | CoCH9 | | N/A | N/A | N/A | 32.41 | 0.21110 |
| GAPDH | CoPL11 | | N/A | N/A | N/A | 31.56 | 0.08540 |
| GAPDH | CoPL14 | | N/A | N/A | N/A | 31.05 | 0.32749 |
| GAPDH | CoPL17 | | N/A | N/A | N/A | 31.43 | 0.07055 |
| MYC | 10%FBS | * | 1.00000 | 0.28418 | 0.28418 | 27.43 | 0.15635 |
| MYC | CoCH15 | | 12.36585 | 2.80227 | 2.80227 | 23.52 | 0.00050 |
| MYC | CoCH16 | | 16.25428 | 2.02311 | 2.02311 | 23.24 | 0.17197 |
| MYC | CoCH9 | | 28.12681 | 8.08900 | 8.08900 | 22.94 | 0.28814 |
| MYC | CoPL11 | | 11.11989 | 0.86103 | 0.86103 | 23.43 | 0.03923 |
| MYC | CoPL14 | | 11.08343 | 4.50910 | 4.50910 | 22.93 | 0.36055 |
| MYC | CoPL17 | | 8.17717 | 1.16099 | 1.16099 | 23.74 | 0.17889 |
| P16 | 10%FBS | * | 1.00000 | 0.37152 | 0.37152 | 27.84 | 0.00000 |
| P16 | CoCH15 | | 21.00160 | 4.76328 | 4.76328 | 23.16 | 0.01347 |
| P16 | CoCH16 | | 19.66719 | 3.19643 | 3.19643 | 23.37 | 0.22871 |
| P16 | CoCH9 | | 29.17628 | 14.61015 | 14.61015 | 23.30 | 0.65787 |
| P16 | CoPL11 | | 15.97940 | 1.77216 | 1.77216 | 23.31 | 0.12108 |
| P16 | CoPL14 | | 8.89957 | 5.40886 | 5.40886 | 23.65 | 0.74453 |
| P16 | CoPL17 | | 20.58880 | 18.04828 | 18.04828 | 22.82 | 1.26073 |
| P21 | 10%FBS | * | 1.00000 | 0.26973 | 0.26973 | 28.19 | 0.08826 |
| P21 | CoCH15 | | 0.35612 | 0.08302 | 0.08302 | 29.39 | 0.07892 |
| P21 | CoCH16 | | 0.32623 | 0.02556 | 0.02556 | 29.63 | 0.10051 |
| P21 | CoCH9 | | 212.39376 | 87.48629 | 87.48629 | 20.78 | 0.51382 |
| P21 | CoPL11 | | 0.46219 | 0.03372 | 0.03372 | 28.77 | 0.01165 |
| P21 | CoPL14 | | 0.43032 | 0.14482 | 0.14482 | 28.37 | 0.14575 |
| P21 | CoPL17 | | 123.66350 | 8.95672 | 8.95672 | 20.58 | 0.03104 |
| P27 | 10%FBS | * | 1.00000 | 0.46442 | 0.46442 | 34.63 | 0.55252 |
| P27 | CoCH15 | | 0.13720 | 0.04397 | 0.04397 | 37.21 | 0.00000 |
| P27 | CoCH16 | | 1.47114 | 0.07452 | 0.07452 | 33.90 | 0.00000 |
| P27 | CoCH9 | | 1.23842 | 0.47147 | 0.47147 | 34.65 | 0.46103 |
| P27 | CoPL11 | | 0.25274 | 0.02591 | 0.02591 | 36.09 | 0.00000 |
| P27 | CoPL14 | | N/A | N/A | N/A | N/A | N/A |
| P27 | CoPL17 | | 1.65291 | 0.16167 | 0.16167 | 33.25 | 0.00000 |
| SNAI1 | 10%FBS | * | 1.00000 | 0.37152 | 0.37152 | 35.28 | 0.00000 |

| | | | | | | | |
|---|---|---|---|---|---|---|---|
| SNAI1 | CoCH15 | | 37.68033 | 23.74015 | 23.74015 | 29.76 | 0.84813 |
| SNAI1 | CoCH16 | | 12.11937 | 12.63337 | 12.63337 | 31.51 | 1.50299 |
| SNAI1 | CoCH9 | | 48.32494 | 26.50824 | 26.50824 | 30.01 | 0.73291 |
| SNAI1 | CoPL11 | | 15.12221 | 2.77642 | 2.77642 | 30.84 | 0.24335 |
| SNAI1 | CoPL14 | | 14.31208 | 6.64130 | 6.64130 | 30.41 | 0.48341 |
| SNAI1 | CoPL17 | | 18.40371 | 3.89004 | 3.89004 | 30.42 | 0.28816 |
| TWIST | 10%FBS | * | 1.00000 | 0.22226 | 0.22226 | 27.45 | 0.08402 |
| TWIST | CoCH15 | | 5.28508 | 1.90126 | 1.90126 | 24.76 | 0.40308 |
| TWIST | CoCH16 | | 5.92467 | 2.42345 | 2.42345 | 24.71 | 0.58786 |
| TWIST | CoCH9 | | 5.77016 | 1.49852 | 1.49852 | 25.24 | 0.22639 |
| TWIST | CoPL11 | | 3.56577 | 1.01240 | 1.01240 | 25.09 | 0.39603 |
| TWIST | CoPL14 | | 2.21929 | 0.72401 | 0.72401 | 25.26 | 0.08383 |
| TWIST | CoPL17 | | 1.92014 | 0.25377 | 0.25377 | 25.85 | 0.16248 |

Inter-run Calibration

MYC

P16

GAPDH

1-SYBR vs. 3-SYBR

Inter-run Calibration

| Inter-run Calibration | 1-SYBR | 3-SYBR | ΔCq | Inter-run Calibration | 1-SYBR | 3-SYBR | ΔCq | Inter-run Calibration | 1-SYBR | 3-SYBR | ΔCq | Inter-run Calibration | 1-SYBR | 3-SYBR | ΔCq | Inter-run Calibration | 1-SYBR | 3-SYBR | ΔCq |
|---|---|---|---|---|---|---|---|---|---|---|---|---|---|---|---|---|---|---|---|
| CoPL17 | 31.3661 | 32.5475 | -1.1814 | | | | | | | | | | | | | | | | |
| 10%FBS | 32.7014 | 32.8377 | -0.1364 | | | | | | | | | | | | | | | | |
| CoCH9 | 32.0046 | 33.6028 | -1.5982 | | | | | | | | | | | | | | | | |
| CoPL14 | 30.5236 | 32.6402 | -2.1166 | | | | | | | | | | | | | | | | |
| CoCH15 | 32.0644 | 32.3218 | -0.2574 | | | | | | | | | | | | | | | | |
| Average ΔCq: -1.0580 | | | | | | | | | | | | | | | | | | | |
| Average ΔΔCq: 1.9802 | | | | | | | | | | | | | | | | | | | |

SNAI1

CyclinD1

TWIST

1-SYBR vs. 2-SYBR

Inter-run Calibration

| Inter-run Calibration | 1-SYBR | 2-SYBR | ΔCq |
|---|---|---|---|
| 10%FBS | 27.4491 | 31.3227 | -3.8736 |
| Average ΔCq: -3.8736 | | | |
| Average ΔΔCq: 0.0000 | | | |

P21
