# Supplementary material for: Human Mesenchymal Stem Cells Derived from the Placenta and Chorion Suppress the Proliferation while Enhancing the Migration of Human Breast Cancer Cells
Source: Stem Cells Int. 2022 Nov 11;2022:4020845. doi: 10.1155/2022/4020845 (PMC9674426; doi:10.1155/2022/4020845)
Supplement: Supplementary Materials — Table S1. Effect of hMSCs on MCF-7 gene expression.pdf which shows the expression level of genes in MCF-7 cells cocultured with hMSCs. Table S2. Effect of hMSCs on MB231 gene expression.pdf which shows the expression level of genes in MDA-MB231 cells cocultured with hMSCs. Table S3. Effect of hMSCs on MCF7 gene expression raw qRT-PCR data as exported from the Bio-Rad.mgxd file. Table S4. Effect of hMSCs on raw qRT-PCR data for MB231 gene expression exported from the Bio-Rad.mgxd file. [file 4020845.f1.zip › Effect of hMSCs on MB231 gene expression.pdf]

Table format: Grouped		Group A		Group B		Grou
		10%FBS		coCH15		coC
		Mean	SEM	Mean	SEM	Mean
1	CyclinD1	1.00000	0.28976	1.26531	0.28762	0.78424
2	E2F2	1.00000	0.87750	1.71572	0.91681	0.64584
3	MYC	1.00000	0.28418	12.36585	2.80227	16.25428
4	P16	1.00000	0.37152	21.00160	4.76328	19.66719
5	P21	1.00000	0.26973	0.35612	0.08302	0.32623
6	P27	1.00000	0.46442	0.13720	0.04397	1.47114
7	SNAI1	1.00000	0.37152	37.68033	23.74015	12.11937
8	TWIST	1.00000	0.22226	5.28508	1.90126	5.92467

	up C	Group D		Group E		Grou
	H16	coCH9		coPL11		coP
	SEM	Mean	SEM	Mean	SEM	Mean
1	0.10344	3.79400	0.78806	1.33768	0.09877	0.32805
2	0.02353	0.40844	0.19194	1.91844	0.53292	1.35467
3	2.02311	28.12681	8.08900	11.11989	0.86103	11.08343
4	3.19643	29.17628	14.61015	15.97940	1.77216	8.89957
5	0.02556	212.39376	87.48629	0.46219	0.03372	0.43032
6	0.07452	1.23842	0.47147	0.25274	0.02591	1.00000
7	12.63337	48.32494	26.50824	15.12221	2.77642	14.31208
8	2.42345	5.77016	1.49852	3.56577	1.01240	2.21929

	up F	Group G	
	L14	coPL17	
	SEM	Mean	SEM
1	0.10611	1.28274	0.14276
2	0.45234	1.00000	0.00000
3	4.50910	8.17717	1.16099
4	5.40886	20.58880	18.04828
5	0.14482	123.66350	8.95672
6	0.00000	1.65291	0.16167
7	6.64130	18.40371	3.89004
8	0.72401	1.92014	0.25377
