# Supplementary material for: Human Mesenchymal Stem Cells Derived from the Placenta and Chorion Suppress the Proliferation while Enhancing the Migration of Human Breast Cancer Cells
Source: Stem Cells Int. 2022 Nov 11;2022:4020845. doi: 10.1155/2022/4020845 (PMC9674426; doi:10.1155/2022/4020845)
Supplement: Supplementary Materials — Table S1. Effect of hMSCs on MCF-7 gene expression.pdf which shows the expression level of genes in MCF-7 cells cocultured with hMSCs. Table S2. Effect of hMSCs on MB231 gene expression.pdf which shows the expression level of genes in MDA-MB231 cells cocultured with hMSCs. Table S3. Effect of hMSCs on MCF7 gene expression raw qRT-PCR data as exported from the Bio-Rad.mgxd file. Table S4. Effect of hMSCs on raw qRT-PCR data for MB231 gene expression exported from the Bio-Rad.mgxd file. [file 4020845.f1.zip › Effect of hMSCs on MCF7 gene expression raw qRT-PCR data.pdf]

hMSC-MCF7 complete.mgxd

10/21/2022 2:33 PM

Report Information

User : BioRad/admin
File Name : hMSC-MCF7 complete.mgxd
File Path : \\Mac\Home\Desktop\SCI re-submission 2022\Gene expression data
Report Differs from Last Save : No

Gene Study File List

File Name	File Path	Date Created	Well Group Name	Run Type	Protocol Edited
2021-08-12 Experiment2.MCF7 target gene part1.pcrd	\\Mac\Home\Desktop	8/12/2021 3:03:32 PM	All Wells	User-defined	No
MCF7 5R.pcrd	\\Mac\Home\Desktop	11/9/2021 5:38:32 PM	All Wells	User-defined	No
2021-11-03 MCF7 4R result.pcrd	\\Mac\Home\Desktop	11/3/2021 8:09:34 PM	All Wells	User-defined	No
2021-10-11 Experiment 7 MCF7 target gene part 3R.pcrd	\\Mac\Home\Desktop	10/11/2021 8:22:39 PM	All Wells	User-defined	No

Study Analysis - Bar Chart

Analysis Mode : Normalized expression (ΔΔCq)
Chart Data : Relative to control
Scaling options :
Chart Error : ± 1.0 SEMs

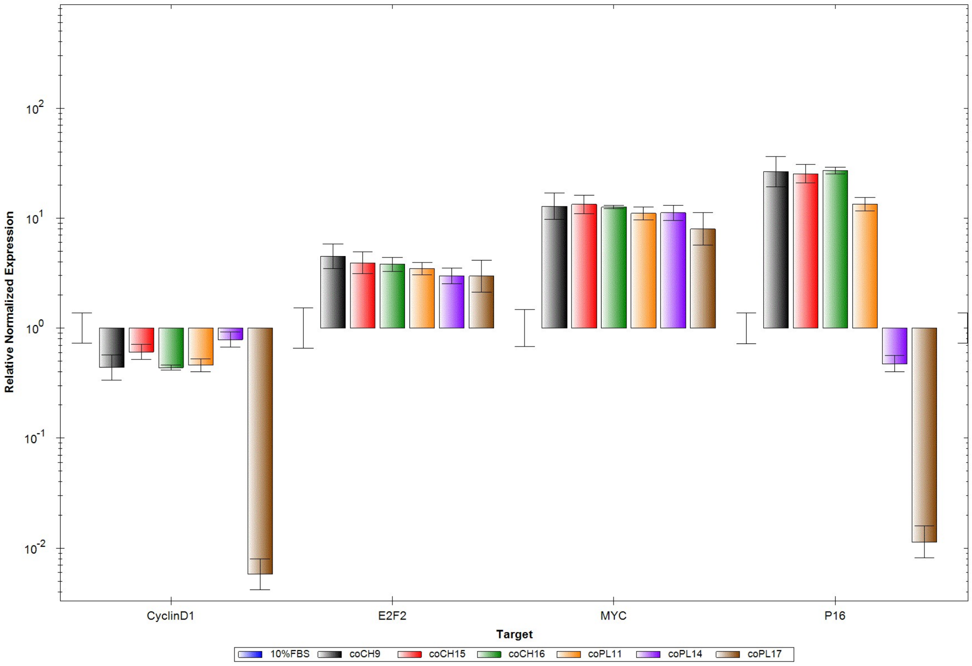

Target Names

Name	Full Name	Reference	Auto Efficiency	Efficiency
CyclinD1	CyclinD1	False	Yes	100.0%
E2F2	E2F2	False	Yes	100.0%
MYC	MYC	False	Yes	100.0%
P16	P16	False	Yes	100.0%
P21	P21	False	Yes	100.0%
P27	P27	False	Yes	100.0%
SNAI1	SNAI1	False	Yes	100.0%
TWIST	TWIST	False	Yes	100.0%
GAPDH	GAPDH	True	Yes	100.0%

Sample Names

Name	Full Name	Control
10%FBS	10%FBS	Yes
coCH9	coCH9	No

coCH15	coCH15	No
coCH16	coCH16	No
coPL11	coPL11	No
coPL14	coPL14	No
coPL17	coPL17	No

Study Analysis - Bar Chart Data

Target	Sample	Ctrl	Expression	Expression SEM	Corrected Expression SEM	Mean Cq	Cq SEM
CyclinD1	10%FBS	*	1.00000	0.37294	0.37294	17.08	0.03588
CyclinD1	coCH15		0.60694	0.10531	0.10531	17.06	0.12530
CyclinD1	coCH16		0.43722	0.02408	0.02408	17.51	0.06677
CyclinD1	coCH9		0.43874	0.13034	0.13034	17.53	0.00846
CyclinD1	coPL11		0.45918	0.06448	0.06448	17.21	0.04711
CyclinD1	coPL14		0.78655	0.13744	0.13744	16.63	0.07067
CyclinD1	coPL17		0.00578	0.00222	0.00222	23.30	0.07737
E2F2	10%FBS	*	1.00000	0.52094	0.52094	29.98	0.52598
E2F2	coCH15		3.92449	1.00355	1.00355	27.26	0.29857
E2F2	coCH16		3.79887	0.59561	0.59561	27.29	0.22206
E2F2	coCH9		4.48560	1.33900	1.33900	27.07	0.04290
E2F2	coPL11		3.45974	0.48276	0.48276	27.19	0.04129
E2F2	coPL14		2.98673	0.53091	0.53091	27.60	0.08487
E2F2	coPL17		2.96645	1.16939	1.16939	27.20	0.15569
GAPDH	10%FBS	*	N/A	N/A	N/A	34.40	0.37960
GAPDH	coCH15		N/A	N/A	N/A	33.66	0.13705
GAPDH	coCH16		N/A	N/A	N/A	33.64	0.03043
GAPDH	coCH9		N/A	N/A	N/A	33.66	0.30301
GAPDH	coPL11		N/A	N/A	N/A	33.40	0.12461
GAPDH	coPL14		N/A	N/A	N/A	33.61	0.15305
GAPDH	coPL17		N/A	N/A	N/A	33.19	0.34595
MYC	10%FBS	*	1.00000	0.46727	0.46727	31.42	0.40775
MYC	coCH15		13.32565	2.77534	2.77534	26.94	0.20815
MYC	coCH16		12.62193	0.41496	0.41496	27.00	0.01993
MYC	coCH9		12.80815	4.07692	4.07692	27.00	0.16508
MYC	coPL11		11.08864	1.59510	1.59510	26.95	0.06518
MYC	coPL14		11.19485	1.88986	1.88986	27.14	0.02746
MYC	coPL17		7.95197	3.23066	3.23066	27.22	0.21058
P16	10%FBS	*	1.00000	0.38154	0.38154	31.91	0.12162
P16	coCH15		25.24049	5.39904	5.39904	26.50	0.21972
P16	coCH16		27.12808	1.89514	1.89514	26.38	0.09113
P16	coCH9		26.41283	9.96220	9.96220	26.44	0.33536
P16	coPL11		13.37708	2.05318	2.05318	27.16	0.10105
P16	coPL14		0.47331	0.08789	0.08789	32.19	0.11492
P16	coPL17		0.01135	0.00451	0.00451	37.16	0.17063
P21	10%FBS	*	1.00000	0.37465	0.37465	28.46	0.06287
P21	coCH15		0.30594	0.06964	0.06964	29.42	0.24679
P21	coCH16		0.23685	0.04968	0.04968	29.77	0.29955
P21	coCH9		0.32838	0.09821	0.09821	29.32	0.05043
P21	coPL11		0.22787	0.03301	0.03301	29.59	0.06963
P21	coPL14		0.34400	0.05783	0.05783	29.20	0.01638
P21	coPL17		19.39579	8.75256	8.75256	22.97	0.35305
P27	10%FBS	*	1.00000	0.52624	0.52624	35.68	0.00000
P27	coCH15		0.49525	0.10898	0.10898	35.95	0.23201
P27	coCH16		0.35335	0.01491	0.01491	36.42	0.00000
P27	coCH9		0.37905	0.18274	0.18274	36.34	0.54785
P27	coPL11		0.09574	0.01849	0.01849	38.07	0.00000
P27	coPL14		0.36158	0.06067	0.06067	36.35	0.00522
P27	coPL17		0.25615	0.13735	0.13735	36.43	0.00000
SNAI1	10%FBS	*	1.00000	0.37636	0.37636	35.68	0.08140
SNAI1	coCH15		4.80999	1.50824	1.50824	32.66	0.39710
SNAI1	coCH16		4.43720	2.94181	2.94181	32.77	0.95552
SNAI1	coCH9		2.86950	1.60055	1.60055	33.42	0.68112
SNAI1	coPL11		3.73349	0.93448	0.93448	32.78	0.30261
SNAI1	coPL14		3.55562	0.76551	0.76551	33.05	0.19472
SNAI1	coPL17		2.71424	1.05141	1.05141	33.03	0.11452
TWIST	10%FBS	*	1.00000	0.41892	0.41892	36.27	0.27762
TWIST	coCH15		1.15683	0.31431	0.31431	35.31	0.32664
TWIST	coCH16		414.87140	71.31567	71.31567	26.81	0.24423
TWIST	coCH9		355.05044	131.77229	131.77229	27.06	0.32104
TWIST	coPL11		298.16394	40.92703	40.92703	27.05	0.01988
TWIST	coPL14		0.62852	0.10547	0.10547	36.14	0.00658
TWIST	coPL17		194.35082	80.91115	80.91115	27.46	0.24807

Inter-run Calibration

GAPDH

1-SYBR vs. 3-SYBR

Inter-run Calibration

Inter-run Calibration	1-SYBR	3-SYBR	ΔCq	Inter-run Calibration	1-SYBR	3-SYBR	ΔCq	Inter-run Calibration	1-SYBR	3-SYBR	ΔCq	Inter-run Calibration	1-SYBR	3-SYBR	ΔCq	Inter-run Calibration	1-SYBR	3-SYBR	ΔCq	Inter-run Calibration	1-SYBR	3-SYBR	Δ[illegible]
10%FBS	34.0943	34.8935	-0.7991																				
coPL11	33.3147	33.7195	-0.4047																				
coPL14	33.6739	33.6879	-0.0141																				
coPL17	33.3265	33.1731	0.1534																				
coCH9	34.0760	33.4326	0.6434																				
coCH15	33.4447	34.1561	-0.7114																				
coCH16	33.6579	33.8083	-0.1504																				
Average ΔCq: -0.1833																							
Average ΔΔCq: 1.4425																							

MYC

P16

TWIST

P21
