# Supplementary material for: Human Mesenchymal Stem Cells Derived from the Placenta and Chorion Suppress the Proliferation while Enhancing the Migration of Human Breast Cancer Cells
Source: Stem Cells Int. 2022 Nov 11;2022:4020845. doi: 10.1155/2022/4020845 (PMC9674426; doi:10.1155/2022/4020845)
Supplement: Supplementary Materials — Table S1. Effect of hMSCs on MCF-7 gene expression.pdf which shows the expression level of genes in MCF-7 cells cocultured with hMSCs. Table S2. Effect of hMSCs on MB231 gene expression.pdf which shows the expression level of genes in MDA-MB231 cells cocultured with hMSCs. Table S3. Effect of hMSCs on MCF7 gene expression raw qRT-PCR data as exported from the Bio-Rad.mgxd file. Table S4. Effect of hMSCs on raw qRT-PCR data for MB231 gene expression exported from the Bio-Rad.mgxd file. [file 4020845.f1.zip › Effect of hMSCs on MCF-7 gene expression.pdf]

Table format: Grouped		Group A		Group B	
		10%FBS		coCH15	
		Mean	SEM	Mean	SEM
1	CyclinD1	1.00000	0.37294	0.60694	0.10531
2	P16	1.00000	0.38154	25.24049	5.39904
3	P21	1.00000	0.37465	0.30594	0.06964
4	P27	1.00000	0.52624	0.49525	0.10898
5	MYC	1.00000	0.46727	13.32565	2.77534
6	SNAI1	1.00000	0.37636	4.80999	1.50824
7	TWIST	1.00000	0.41892	1.15683	0.31431

	Group C		Group D		Grou[illegible]
	coCH16		coCH9		coP[illegible]
	Mean	SEM	Mean	SEM	Mean
1	0.43722	0.02408	0.43874	0.13034	0.45918
2	27.12808	1.89514	26.41283	9.96220	13.37708
3	0.23685	0.04968	0.32838	0.09821	0.22787
4	0.35335	0.01491	0.37905	0.18274	0.09574
5	12.62193	0.41496	12.80815	4.07692	11.08864
6	4.43720	2.94181	2.86950	1.60055	3.73349
7	414.87140	71.31567	355.05044	131.77229	298.16394

	up E	Group F		Group G	
	L11	coPL14		coPL17	
	SEM	Mean	SEM	Mean	SEM
1	0.06448	0.78655	0.13744	0.00578	0.00222
2	2.05318	0.47331	0.08789	0.01135	0.00451
3	0.03301	0.34400	0.05783	19.39579	8.75256
4	0.01849	0.36158	0.06067	0.25615	0.13735
5	1.59510	11.19485	1.88986	7.95197	3.23066
6	0.93448	3.55562	0.76551	2.71424	1.05141
7	40.92703	0.62852	0.10547	194.35082	80.91115
